# Jingui Shenqi pill for nocturia due to nocturnal polyuria: A protocol for systematic review and meta-analysis

**DOI:** 10.1097/MD.0000000000023742

**Published:** 2020-12-18

**Authors:** Zhipeng Fan, Zhaodi Wang, Jiashuai Deng, Yong Jiang

**Affiliations:** Basic Medical College, Chengdu University of TCM, Chengdu, Sichuan Province, China.

**Keywords:** Jingui Shenqi pill, effectiveness, safety, nocturia, nocturnal polyuria, systematic review

## Abstract

**Background:**

The incidence of nocturia is high and will seriously affect patients’ physical and mental health. Nocturnal polyuria is the most critical cause of nocturia, There are few drugs currently used to treat nocturia due to Nocturnal Polyuria (NP). The guide highly recommends only Desmopressin. There is an urgent need to find new drugs. Jingui Shenqi pill (JSP) is a Chinese patent medicine, it is widely used in China to treat NP. However, there is no evidence-based medical evidence to prove its safety and effectiveness. The purpose of this systematic review is to evaluate the efficacy and safety of JSP in the treatment of NP.

**Methods:**

We will search the randomized controlled trials (RCTs) including JSP for NP and published from the inception of the database to Oct 2020 by the following eight databases: Embase, Cochrane Library, PubMed, MEDLINE, the China National Knowledge Infrastructure, Cqvip Database, and Wanfang Data, the Chinese Biomedical Literature Database. There is no language and publication status restriction. The primary outcomes will include Nocturnal urine volume, the number of nocturnal voids, Nocturnal polyuria index (Nocturnal total urine volume /24 h total urine volume). We will perform the data synthesis, sensitivity analysis, subgroup analysis, and bias assessment risk using RevMan V.5.3. The reporting bias will be assessed using a funnel plot and Egger test.

**Results:**

This study may provide additional evidence of JSP for NP in the effectiveness and safety and alternative therapy for NP.

**Conclusions:**

In this systematic review, we will assess whether JSP is an effective and safe medicine for nocturia.

## 1 Introduction

Nocturia is defined as the patient must wake up one or more times to void at night.^[1]^ It is generally believed that being awake at night to void two or more times has clinical significance and will severely impact patients’ quality of life.^[2,3]^ nocturia is very common, and the incidence of nocturia is high among adults, especially the elderly. Studies have reported that the prevalence of clinically relevant nocturia (≥2 voids per night) in young men aged 20 to 40 years is 2% to 16.6%. The prevalence rate of young women is 4.4% to 18%. The prevalence rate of older men than 70 years is 29% to 59.3%. In older women than 70 years, the incidence of nocturia is 28.3% to 61.5%.^[4]^ Studies have shown that nocturia can seriously affect patients’ health-related quality of life (HRQL). It will increase the risk of depression, cardiovascular and cerebrovascular events, falls and fractures, and mortality.^[5–9]^

Nocturia has brought a severe socioeconomic burden. A study showed that only the hospitalization cost for a hip fracture caused by nocturia in the EU in 2014 was €1.0 billion. In the United States, the loss of falls due to nocturia alone amounts to about $1.5 billion each year.^[10]^

The leading causes of nocturia are divided into the following categories: nocturnal polyuria, global polyuria, reduced bladder capacity, sleep disorders, and circadian clock disorders. Christoph Klingler et al have found that nocturnal polyuria is the leading cause of nocturia.^[11]^

At present, there are few drugs for NP. According to the guidelines, the only drug highly recommended to treat NP is Desmopressin, which increases the risk of hyponatremiahighly recommended.^[12,13]^ there is a medical need for a new medicine for NP.

JSP is a Chinese patent medicine for NP. It has been clinically used for more than two thousand years. It was first recorded in *the Synopsis of Prescriptions of the Golden Chamber*. It consists of the following herbs: Moutan Cortex (Mu DanPi), Aliamria Rhizoma (Ze Xie), Cinnamomi Ramulus (Gui Zhi), Achyranthis Bidentatae Radix (Niu Xi), Rehmanniae Radix (Di Huang), Dioscoreae Rhizoma (Shan Yao), Poria (Fu Ling), Corni Fructus (Shan Zhu Yu), Plantaginis Semen (Che Qian Zi), and Aconiti Lateralis Rddix Praeparata (Fu Zi).^[14]^ Many modern studies have shown that it is effective in treating NP.^[15–19]^, but due to the small sample, size, it is challenging to get reliable conclusions. This study aims to Provides evidence-based medical evidence for clinical use of JSP as an alternative therapy for NP by identifying and critically evaluate Randomized Controlled Trials (RCTs) of JSP for Treating NP.

## 2 Methods

### 2.1 Study registration

The protocol for this systematic review was registered on INPLASY (10.37766/inplasy2020.11.0048) and is available in full on the inplasy.com (https://doi.org/10.37766/inplasy2020.11.0048)."

The protocol will be strictly implemented under the Preferred Reporting Items for Systematic Reviews and Meta-Analyses Statement (PRISMA-P).

### 2.2 Ethics and dissemination

Ethical approval is not required as we need not collect primary data. The final results of the study will be published in a peer-reviewed journal.

### 2.3 Eligibility criteria

#### 2.3.1 Types of studies

All RCTs about JSP for NP will be included regardless of language. The following studies will be excluded: animal experiments, case series, quasi-RCTs, cell experiments, Case reports, non-RCTs.

#### 2.3.2 Participants

Patients diagnosed with NP and aged > 18 years old will be included, regardless of gender, economic status, or restrictions.

#### 2.3.3 Types of interventions

The intervention group used only JSP or combined JSP with another active treatment, pharmacological or non-pharmacological. The control group used another active treatment or placebo or no treatment. The dosage and frequency will not be restricted.

#### 2.3.4 Types of outcome measures

The primary outcomes include Nocturnal urine volume, the number of nocturnal voids, Nocturnal polyuria index (Nocturnal total urine volume /24 h total urine volume). Secondary outcomes include the time to first void, 24 hours total urine volume, 24 hours total number of voiding

### 2.4 Exclusion criteria

We will exclude the following studies: studies where complete data are not available; studies with data errors; studies using wrong intervention methods or random methods. For duplicate documents, we will select only one of them.

### 2.5 Search strategy

The following eight electronic databases, including PubMed, Cochrane Library, EMBASE, MEDLINE, the China National Knowledge Infrastructure, the Chinese Biomedical Literature Database, Cqvip Database, and Wanfang Data, will be comprehensively searched. All the documents retrieved are from the time of establishing the database to Oct 10, 2020. We will use the following terms to search. “nocturia,” “nycturia,” “noctur,” “nocturnal polyuria,” “nocturnal urine production,” “nocturnal urine volume,” “Jinkui Shenqi pill,” “randomized clinical trials,” and “RCTs.” To identify additional references, we will manually search the reference lists of primary studies and related reviews. We also will search the following resources to identify ongoing or completed clinical trials: Google scholar, Opengrey, Chinese Clinical Trial Registry, ClinicalTrials.gov, International Clinical Trials Registry Platform.

### 2.6 Study selection and data extraction

#### 2.6.1 Study selection

**Figure 1:**
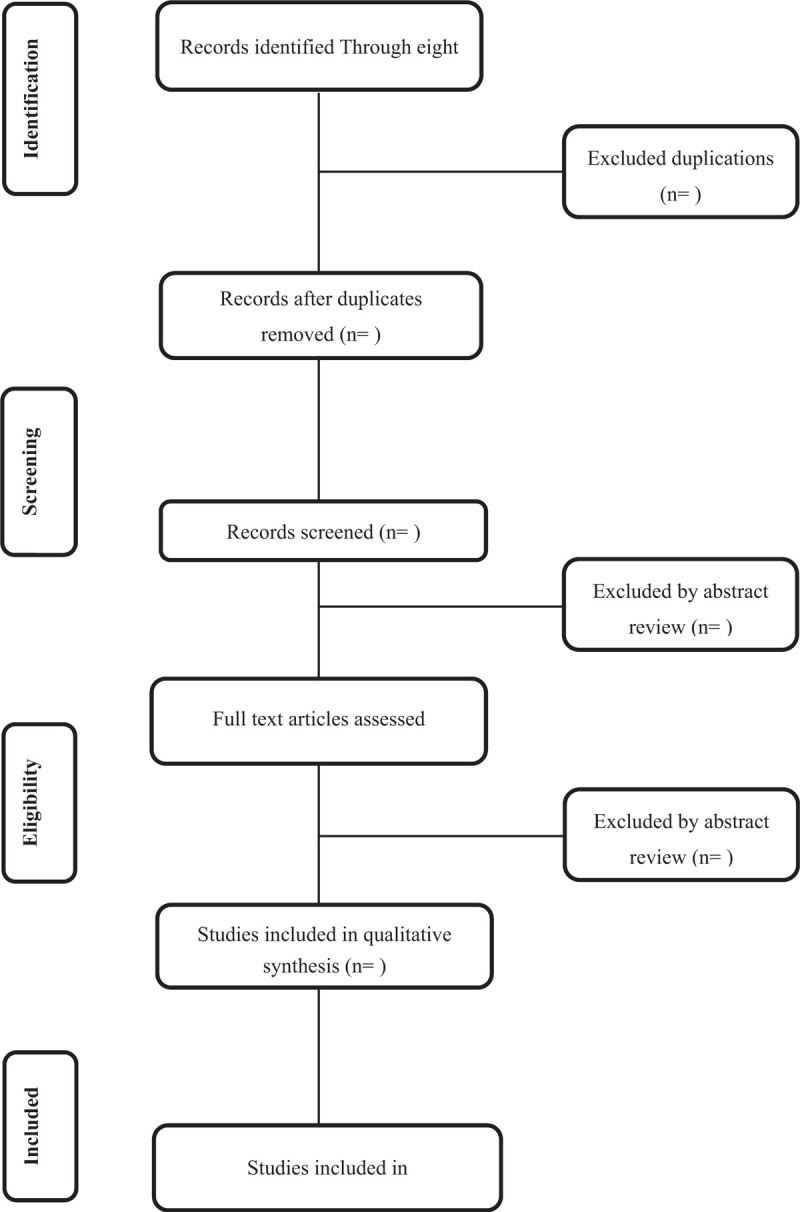
Flow diagram of the study selection process.

We will use EndNote X7 to manage literature and remove duplications. The two reviewers will individually screen out potential eligible studies based on the search strategy. The two researchers will carefully read the title and abstract to eliminate irrelevant studies and then read the full text to confirm eligible studies. If any dispute occurs, the dispute will be resolved through consensus or consultation with the third reviewer. The process will be shown through the PRISMA flow diagram (Fig. 1).^[20]^

#### 2.6.2 Data extraction

Data extraction is fulfilled by two researchers independently. Data extracted include

(a) Literatures information (title, author, year of publication, country);
(b) Study characteristics (Study design, details of randomization process, sample size, inclusion and exclusion criteria);
(c) Participant characteristics (gender, age, diagnosis criteria);
(d) intervention details;
(e) Outcomes.

Any disputes about data extraction will be resolved through consensus. If necessary, the study's further details or clarification will be supplemented by contacting the original author.

### 2.7 Assessment of risk of bias

Two reviewers will use the Cochrane Collaboration's tool to assess methodological quality.^[21]^ We will evaluate the following seven aspects. Including random, blinding of participants and investigators, sequence generation, the blindness of outcome assessments, allocation concealment, selective outcome reporting, incomplete outcome data, and other biases. Each included study will be assessed as low, unclear, or high bias based on the results. Any discrepancies will be resolved by further discussing it with a third reviewer.

### 2.8 Statistical analysis

For data analysis, we will use RevMan 5.3.0 provided by the Cochrane Collaboration. The chi-square test and I^2^ statistic will be used to assess the heterogeneity of similar studies. If *P* ≥ .05 and I^2^ ≤ 50%, it is low heterogeneity. As a result, a fixed-effects model will be used. If *P* < .05 and I^2^ > 50%, there will be heterogeneity. a random-effects model will be used. We will use an odds ratio (OR) with a 95% confidence interval (CI) to represent the enumeration data. We will express the measurement data by the mean difference (MD with 95% CI. A statistically significant difference is considered to be *P* < .05.

### 2.9 Subgroup analysis and sensitivity analysis

To seek the potential source of heterogeneity, subgroup analysis, and sensitivity analysis will be performed based on various study characteristics such as study quality, location of study, publication date, trial publishing status, type of comparisons, history of pulmonary diseases, duration of treatment, frequency of delivery, age, gender.

### 2.10 Publication bias

We evaluate if there will is a reporting bias using a funnel plot and the Egger test.

## 3 Discussion

TCM believes that the deficiency of kidney yang causes NP. JSP is a standard medicine used in TCM to treat deficiency of kidney yang. Modern research has found that it can treat NP.^[15,16]^ Because of the remarkable effect, JSP is now widely using to treat NP in China. We hope to obtain an evidence-based medicine that JSP is a safe and effective drug for NP treatment by systematically evaluating the RCTs of NP's treatment using JSP

## Author contributions

**Conceptualization:** Zhipeng Fan, Zhaodi Wang, Jiashuai Deng.

**Data curation:** Yong Jiang.

**Investigation:** Zhaodi Wang.

**Methodology:** Zhipeng Fan, Zhaodi Wang.

**Project administration:** Zhipeng Fan, Jiashuai Deng.

**Software:** Yong Jiang.

**Supervision:** Jiashuai Deng.

**Validation:** Zhaodi Wang.

**Writing – original draft:** Zhipeng Fan.

**Writing – review & editing:** Zhipeng Fan, Zhaodi Wang.

## References

[1] Van Kerrebroeck P Abrams P Chaikin D The standardization of terminology in nocturia: report from the standardization subcommittee of the International Continence Society. BJU Int 2002;90: Suppl 3: 11–5. 12445092 10.1046/j.1464-410x.90.s3.3.x

[2] Weiss JP Blaivas JG Bliwise DL The evaluation and treatment of nocturia: a consensus statement. BJU Int 2011;108:6–21. 21676145 10.1111/j.1464-410X.2011.10175.x

[3] Coyne KS Zhou Z Bhattacharyya SK The prevalence of nocturia and its effect on health-related quality of life and sleep in a community sample in the USA. BJU Int 2003;92:948–54. 14632853 10.1111/j.1464-410x.2003.04527.x

[4] Bosch JL Weiss JP The prevalence and causes of nocturia. J Urol 2013;189: 1 Suppl: S86–92. 23234639 10.1016/j.juro.2012.11.033

[5] Funada S Tabara Y Negoro H Longitudinal analysis of bidirectional relationships between nocturia and depressive symptoms: the nagahama study. J Urol 2020;203:984–90. 31750764 10.1097/JU.0000000000000683

[6] Lightner DJ Krambeck AE Jacobson DJ Nocturia is associated with an increased risk of coronary heart disease and death. BJU Int 2012;110:848–53. 22233166 10.1111/j.1464-410X.2011.10806.x PMC3508707

[7] Kupelian V Fitzgerald MP Kaplan SA Association of nocturia and mortality: results from the Third National Health and Nutrition Examination Survey. J Urol V 185 2011;571–7. 10.1016/j.juro.2010.09.108 21168875

[8] Kim SY Bang W Kim MS Nocturia is associated with slipping and falling 2017;12:e0169690. 10.1371/journal.pone.0169690 PMC5218404 28060916

[9] Pesonen JS Vernooij RWM Cartwright R The impact of nocturia on falls and fractures: a systematic review and meta-analysis. J Urol 2020;203:674–83. 31347956 10.1097/JU.0000000000000459

[10] Holm-Larsen T The economic impact of nocturia. Neurourol Urodyn 2014;33: Suppl 1: S10–4. 24729147 10.1002/nau.22593

[11] Klingler HC Heidler H Madersbacher H Nocturia: an Austrian study on the multifactorial etiology of this symptom. Neurourol Urodyn 2009;28:427–31. 19229953 10.1002/nau.20665

[12] Fralick M Schneeweiss S Desmopressin and the risk of hyponatremia: a population-based cohort study 2019;16:e1002930. 10.1371/journal.pmed.1002930 PMC6802819 31634354

[13] Choi EY Park JS Kim YT The risk of hyponatremia with desmopressin use for nocturnal polyuria. Am J Nephrol 2015;41:183–90. 25871541 10.1159/000381562

[14] Zhao L Zhao A Chen T Global and targeted metabolomics evidence of the protective effect of chinese patent medicine jinkui shenqi pill on adrenal insufficiency after acute glucocorticoid withdrawal in rats. J Proteome Res 2016;15:2327–36. 27267777 10.1021/acs.jproteome.6b00409 PMC5614501

[15] Yu-tong W Lei W Research progress on Jinguishenqi Pill in clinical and pharmacological experiment. Guiding J Trad Chin Medic Pharm 2015;21:53–5.

[16] Chunzhi W Wen Y Fugen G Progress in clinical application of Jinkuishenqi Pills (tablets and decoctions). China Pharm 2014;25:2298–302.

[17] Zhu X Zhu L Clinical observation on treatment of 90 cases of adult nocturia with jinkuishenqi pills. J N Chin Med 2011;43:45–6.

[18] Zuhan Y Jinkui Shenqi Pill (soup) for treatment of nocturia in elderly women. Hubei J Trad Chin Med 2006;37.

[19] Ke C New clinical uses of jinkuishenqi pills. J Sichuan Trad Chin Med 2008;124–5.

[20] Shamseer L Moher D Clarke M Preferred reporting items for systematic review and meta-analysis protocols (PRISMA-P) 2015: elaboration and explanation. BMJ (Clinical research ed) 2015 1 2;350:g7647. 10.1136/bmj.g7647 25555855

[21] Higgins JP Altman DG Gøtzsche PC The Cochrane Collaboration's tool for assessing risk of bias in randomised trials. BMJ (Clinical research ed) 2011 10 18;343:d5928. 10.1136/bmj.d5928 PMC3196245 22008217

